# Optimization of the STARlet workflow for semi-automatic SARS-CoV-2 screening of swabs and deep respiratory materials using the RealAccurate Quadruplex SARS-CoV-2 PCR kit and Allplex SARS-CoV-2 PCR kit

**DOI:** 10.1128/spectrum.03296-23

**Published:** 2024-01-09

**Authors:** Jacky Flipse, Angelino T. Tromp, Danique Thijssen, Nicole van Xanten-Jans-Beken, Roy Pauwelsen, Harmen J. van der Veer, Juliëtte M. Schlaghecke, Caroline M. A. Swanink

**Affiliations:** 1 Laboratory for Medical Microbiology and Immunology, Rijnstate Hospital, Velp, the Netherlands; 2 Laboratory of Chemical Biology, Department of Biomedical Engineering, Eindhoven University of Technology, Eindhoven, the Netherlands; 3 Institute for Complex Molecular Systems, Eindhoven University of Technology, Eindhoven, the Netherlands; 4 Research Group Applied Natural Sciences, Fontys University of Applied Sciences, Eindhoven, the Netherlands; University of Cincinnati, Cincinnati, Ohio, USA

**Keywords:** SARS-CoV-2, laboratorium automation, validation, molecular diagnostics, lean management, turnaround time

## Abstract

The severe acute respiratory syndrome coronavirus 2 (SARS-CoV-2) pandemic triggered the implementation of large-scale screenings in the health care and in the general population. Consequently, medical laboratories have to apply lean laboratory management to design workflows that are able to process large batches within short turnaround times while maintaining flexibility to use different SARS-CoV-2 reverse transcription polymerase chain reactions (RT-PCRs) and to be able to process a variety of clinical samples. We validated two SARS-CoV-2 PCR assays on the STARlet workflow: Allplex SARS-CoV-2 PCR kit and RealAccurate Quadruplex SARS-CoV-2 PCR kit. Furthermore, we optimized and validated the STARlet workflow for semi-automatic screening for SARS-CoV-2 in upper respiratory swabs and deep respiratory materials (sputa, bronchoalveolar lavage, and aspirate). Strikingly, guanidine-containing lysis buffers allow for easy processing and can enhance sensitivity of SARS-COV-2 screening since sampling in these buffers may preserve viral transcripts as evident by the higher copy numbers of the SARS-CoV-2 N gene. Moreover, using the principles of lean laboratory management, several bottlenecks that are typical for medical laboratories were addressed. We show that lean laboratory management resulted in significant reduction of the turnaround times of the SARS-CoV-2 PCR in our laboratory. This report thus describes a useful framework for laboratories to implement similar semi-automated workflows.

**IMPORTANCE:**

The SARS-CoV-2 pandemic triggered the implementation of large-scale screenings in the health care and in the general population. Consequently, medical laboratories had to adapt and evolve workflows that are able to process large batches within short turnaround times while maintaining flexibility to use different assays and to be able to process a variety of clinical samples. We describe how the need for increased outputs and greater flexibility was addressed with respect to clinical samples and assays (Allplex SARS-CoV-2 PCR and RealAccurate Quadruplex SARS-CoV-2 PCR). Strikingly, we found that upper respiratory swabs collected in guanidine-containing lysis buffers both improved the ease of processing as well as enhanced the sensitivity of the SARS-CoV-2 screening. This report thus describes a useful framework for laboratories to implement and optimize similar semi-automated workflows.

## INTRODUCTION

The severe acute respiratory syndrome coronavirus 2 (SARS-CoV-2) pandemic triggered the implementation of large-scale screenings in the health care and in the general population. Consequently, medical laboratories had to evolve workflows that are able to process large batches while reducing turnaround times. At the same time, the sudden increase in demand for consumables and reagents/assays required flexibility within diagnostic chains and laboratories (1, 2).

Technical requirements for SARS-CoV-2 testing vary depending on the population tested, the presence of symptoms, and the phase of disease (3, 4). To meet demands for high capacity, flexibility, and short turnaround times, our laboratory opted to automate the liquid handling using STARlet systems (Hamilton, Reno, Nevada, USA). In this study, we report on the following:

1. The validation of two commercially available assays on the STARlet workflow: Allplex SARS-CoV-2 PCR kit (Seegene, Seoul, South Korea) and RealAccurate Quadruplex (RAQ) SARS-CoV-2 PCR kit (PathoFinder, Maastricht, the Netherlands), which are multiplex assays targeting four [E, N, RNA-dependent RNA polymerase (RdRp), S] and two (N and RdRp) genes, respectively, of SARS-CoV-2.
2. Processing deep respiratory materials (bronchoalveolar lavage, sputa, and bronchial aspirates) and upper respiratory swabs (oropharynx/nasopharynx) in eSwab (Copan, Italy), universal transport medium (UTM, Mediaproducts BV, Groningen, the Netherlands), and PurePrep TL+ lysis buffer (PP-TL+, MolGen, Veenendaal, the Netherlands).
3. Adaptations from the pre-analytical process and middleware system to reduce turnaround times.

## RESULTS

### Validation of semi-automated Allplex SARS-CoV-2 assay

The Allplex SARS-CoV-2 assay has been validated on various workflows (5–7) albeit not on the STARlet workflow. Therefore, we first evaluated the performance of the Allplex SARS-CoV-2 assay on the STARlet workflow against the manual workflow based on a published reverse transcription polymerase chain reaction (RT-PCR) assay (8) using clinical samples and external quality assurance panels (Tables 1 and 2). The Allplex SARS-CoV-2 assay detects SARS-CoV-2 loads at 500 ddPCR (digital droplet PCR) copies/mL and higher, though not in each PCR target at lower loads (Table S1). No cross-reaction was seen between the Allplex SARS-CoV-2 assay and other prevalent respiratory viruses, including endemic coronaviruses (Table S2). However, the E gene RT-PCR cross-reacts with SARS-CoV-1 (Table 2).

**TABLE 1:** Comparison between in-house assay and Allplex SARS-CoV-2 assay on clinical samples

			In-house assay (8)	
			Positive (*n* = 27)	Negative (*n* = 53)
Allplex SARS-CoV-2	UTM (*n* = 19)	Positive	13	
		Negative		6
	eSwab (*n* = 61)	Positive	14	
		Negative		47^*a*^

^*a*^ Seven clinical samples were tested only for SARS-CoV-2. Forty clinical, BioFire RP2.1 plus-positive samples were added for analytical specificity, containing human endemic coronavirus (hCoV) OC43 (*n* = 3), hCoV HKU-1 (*n* = 2), respiratory syncytial virus (*n* = 5), rhinovirus/enterovirus (*n* = 24), adenovirus, unknown type (*n* = 5), influenza virus type A H1pdm09 (*n* = 4), influenza virus type A (H3) (*n* = 2), parainfluenzavirus type 3 (*n* = 2) and type 4 (*n* = 2), human metapneumovirus (*n* = 3), and *Bordetella parapertussis* (*n* = 1). See Table S2 for the overview.

**TABLE 2:** Performance of the Allplex SARS-CoV-2 assay on external quality assurance samples^*d*^

		External quality assurance^*a*^ SARS-CoV-2 loads (ddPCR copies/mL)				
		Unknown	>1 × 10^4^	5 × 10^2^–1 × 10^4^	<5 × 10^2^	Negative
Allplex SARS-CoV-2	Positive	4	36	17	6	5^*b*^
	Negative				4	26^*c*^

^*a*^ External quality assurance (EQA) samples are derived from Quality Control for Molecular Diagnostics (QCMD) and the National Institute for Public Health and the Environment (Bilthoven, the Netherlands). Samples include the following SARS-CoV-2 types: B.1, B.1.1.7; B.1.117; B.1.351; B.1.525; B.1.526; B.1.529 (BA.1; BA.2); B.1.617.2; P.1.

^*b*^ These five SARS-CoV-2-negative samples concern samples containing SARS-CoV-1 and are detected by the sarbeco-broad E gene RT-PCR. Detection of SARS-CoV-1 is recognizable since these samples were only positive for the E gene with a Ct value well below Ct 35, which is highly unusual.

^*c*^ Includes hCoV 229E (*n* = 5); hCoV OC43 (*n* = 6); hCoV NL63 (*n* = 3); influenza virus A type H3N2 (*n* = 4); and true-negative samples (*n* = 8).

^*d*^ The Allplex SARS-CoV-2 assay is a multiplex RT-PCR detecting four SARS-CoV-2 genes with three dyes: E, N, and RdRp and S, with the latter two combined into one dye.

**Fig 1:**
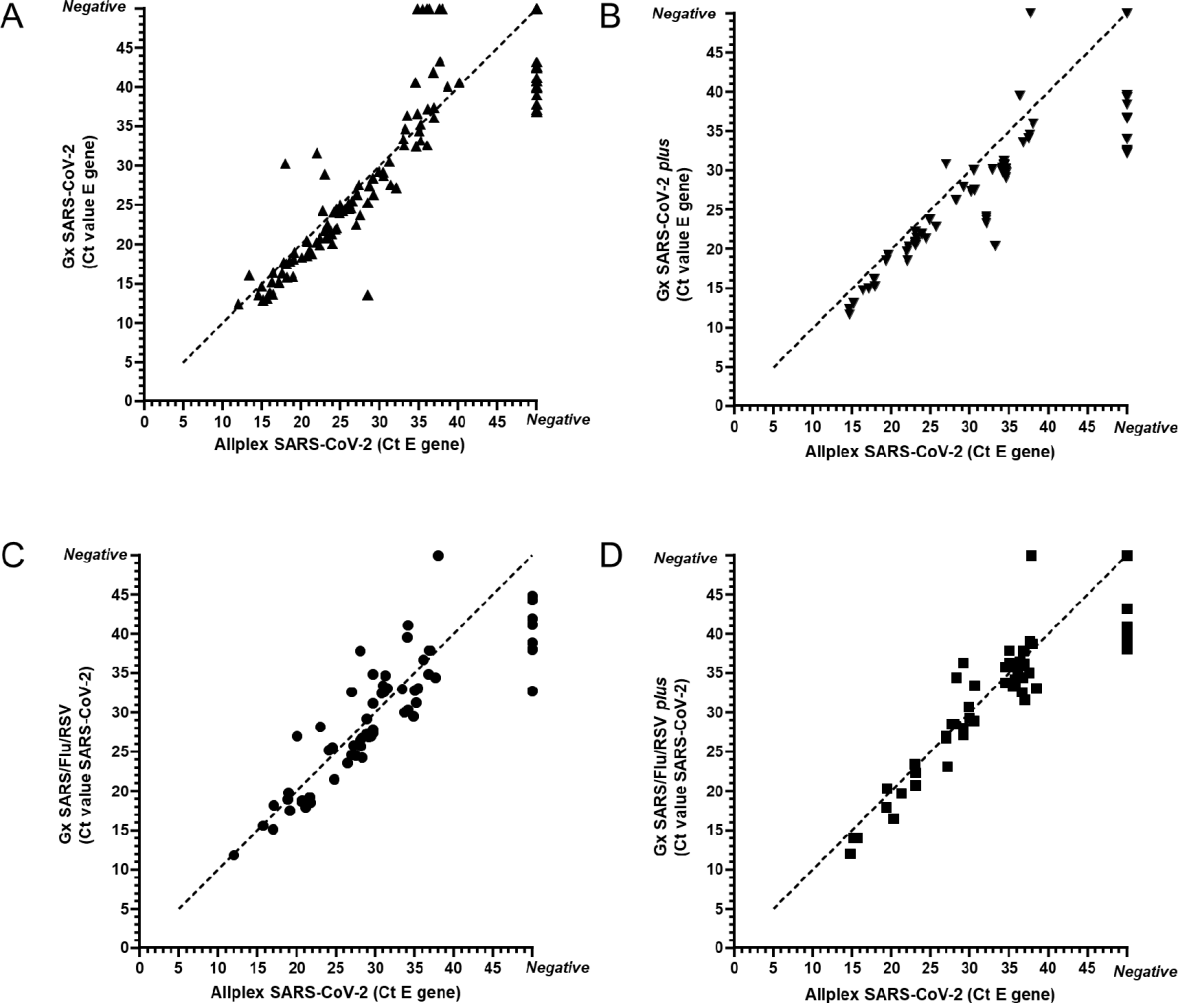
(A–D) Comparison of the Seegene relative to the GeneXpert (Gx) SARS-CoV-2 assays [monoplex SARS-CoV-2 (**A and B**) and SARS/Flu/RSV (**C and D**) in original assay (**A and C**) and with additional RdRp target (**B and D**)]. The dotted line represents the isometric line where both test reports equal Ct values.

Next, we retrospectively and prospectively compared the Allplex SARS-CoV-2 with the GeneXpert SARS-CoV-2 and SARS-CoV-2/Flu/respiratory syncytial virus (RSV) assays on respiratory swabs (9, 10) (Fig. 1A through D; Table S3). Assuming that all SARS-CoV-2 positives are true positive, the sensitivity of the Allplex SARS-CoV-2 assay relative to the various GeneXpert SARS-CoV-2 rapid tests was 89% (108/122; GeneXpert SARS-CoV-2); 89% (56/63; GeneXpert SARS-CoV-2 *plus*), 89% (55/62; GeneXpert SARS-CoV-2/Flu/RSV), and 92% (54/59; GeneXpert SARS-CoV-2/Flu/RSV *plus*), respectively .

Third, carryover studies were performed to evaluate the risk of cross-contamination in the STARlet workflow. Here, seven checkerboard experiments were performed on six apparatuses, comprising a total of 76 high positives (Ct 15–20) and 266 SARS-CoV-2-negative samples. One false positive (N gene Ct 35, negative in E and RdRp/S) was detected. Wipe tests did not find a source of contamination within the apparatus, indicating that this contamination could originate throughout the workflow, from sample loading to sealing the PCR plate.

### Establishing the cutoff for reliable reporting of Allplex SARS-CoV-2 assay results

Finally, we evaluated the internal control (IC) of the Allplex assay. The IC (bacteriophage MS2) is added to the sample and processed and analyzed concurrently with the sample. Thus, the IC verifies proper nucleic acid extraction and RT-PCR amplification, and ensures that the test will be in accordance with ISO15189. The Allplex SARS-CoV-2 assay is considered valid if the IC has a Ct of <42. We wished to define the cutoff for the IC where lower Ct values correspond to reliable PCR results for SARS-CoV-2. IC Ct values above the cutoff are related to inhibition of the PCR and thus potentially non-reliable results. To actively induce PCR inhibition, we added decreasing amounts of heparin, a known PCR inhibitor (11), to our samples. Based on heparin titration, Ct values ≤26 for the IC correspond to non-inhibited PCR reactions (Fig. S1A and B). Indeed, SARS-CoV-2-positive samples where the IC was higher than the IC cutoff showed inefficient PCR amplification resulting in either high or absent Ct values for SARS-CoV-2 targets (Fig. S1B). Moreover, the IC cutoff identified in this experiment corresponded to the mean plus two standard deviations of all IC values seen during routine diagnostics. Thus, the IC cutoff is recalculated for every new lot number of the Allplex SARS-CoV-2 assay.

### Validation of the RAQ SARS-CoV-2 RT-PCR assay

Next, we evaluated the RAQ SARS-CoV-2 RT-PCR kit as an alternative assay and PP-TL+ as an alternative sampling medium. First, the RAQ assay was validated on the STARlet workflow as the chemistry of the STARlet might differ from the NucliSENS easyMag system (12). To demonstrate equivalence of the RAQ and Allplex assays, accordant to EU specifications (13), the limit of detection is 1,050 copies/mL (RAQ, v3 2020) and 5,000 copies/mL (Allplex, Fact Sheet March 2022). Thus, a working solution of a World Health Organization (WHO) SARS-CoV-2 reference strain (5,000 ddPCR copies/mL) is tested on the STARlet workflow in 24-fold (six replicates, each extracted and tested in four independent runs). Both assays achieved 100% detection (24/24) for all SARS-CoV-2 genes, indicating equivalence at this concentration, with high reproducibility (Table S5).

**Fig 2:**
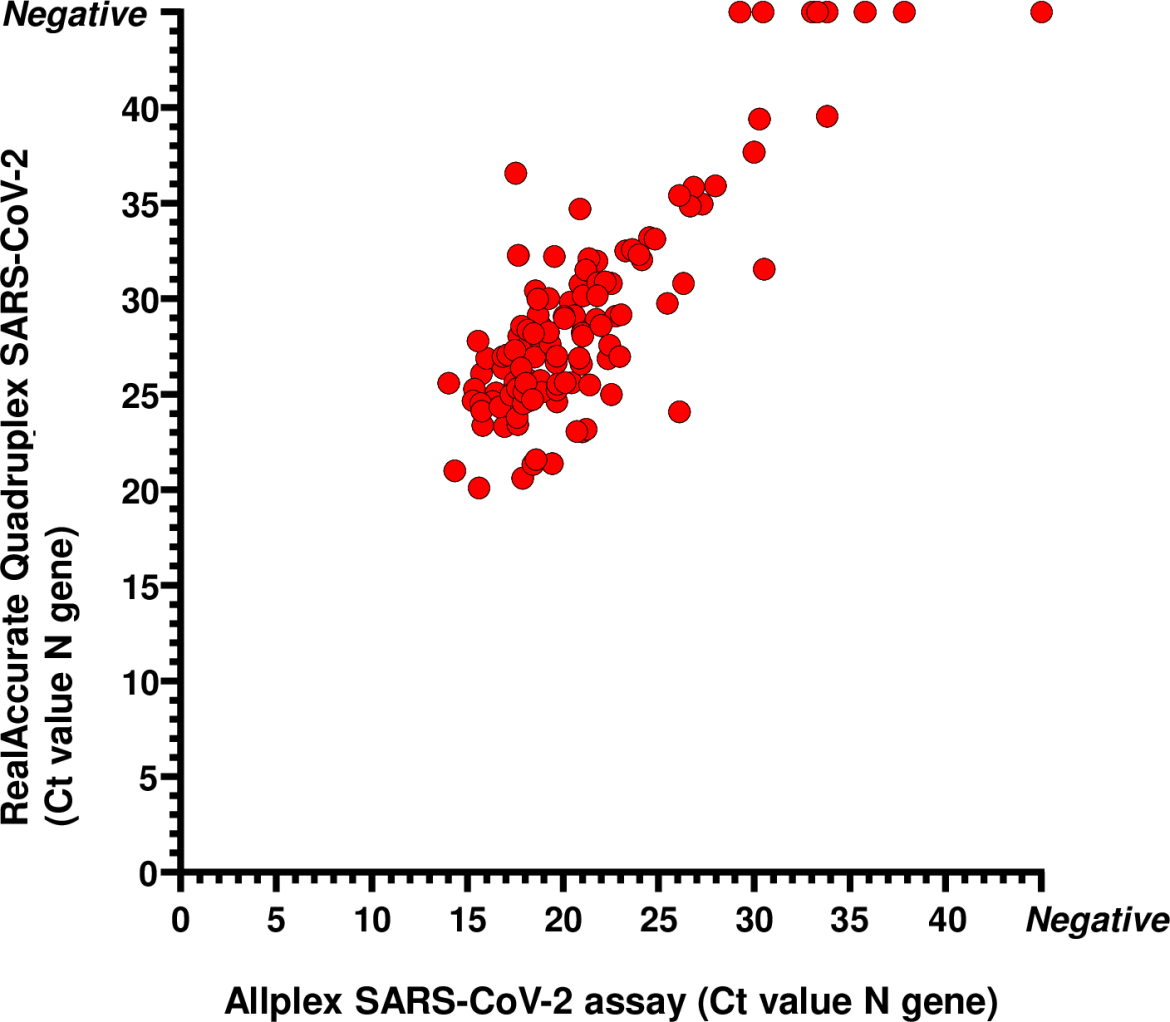
Plot of RAQ SARS-CoV-2 N gene Ct values against Seegene Allplex N gene Ct values. The correlation between the two Ct values is described as 0.9325x + 5.372.

Next, the clinical evaluation was conducted in March–June 2022 and comprised of 121 SARS-CoV-2-positive and 513 SARS-CoV-2-negative samples (Table 3; Fig. 2) (13).

**TABLE 3:** Comparison of the RAQ SARS-CoV-2 assay and the Allplex SARS-CoV-2 assay^*a*^

		Allplex SARS-CoV-2, E gene		
		Ct <35.0 *n* = 117	Ct ≥35.0 *n* = 4	Negative *n* = 513
RAQ SARS-CoV-2	Positive	115	1	0
	Negative	2	3	513

^*a*^ Allplex SARS-CoV-2-positive reports are based on any of the three targets (E gene, N gene, and/or the combined RdRp/S genes). RAQ SARS-CoV-2 assay uses two genes (N and RdRp). All results were N-gene positive. For the RdRp-specific results, see Table S4. None of the samples were invalid due to the presence of inhibitory factors.

In the Netherlands, a guideline was drafted on the possibility of the transmission of SARS-CoV-2 (14), with a cutoff based on the 90th percentile of all RT-PCR-positive cases. This meant that RT-PCR-positive swabs with E gene Ct <35.0 are considered “positive” (“high load”) and those with E-gene Ct ≥35.0 are “weak positive.” Compared to the Allplex SARS-CoV-2 assay, the RAQ assay had a sensitivity of 95.9% (116/121) and a specificity of 100% (513/513). However, if only the “positives” are considered, the sensitivity of the RAQ assay is 98.3% (115/117, Table 3). The relationship between Ct values for the RAQ N gene and the Allplex E gene is described by Ct RAQ N gene = 0.9325x Ct E gene (Allplex) +5.37 (Fig. 2). Overall, the best performance on our workflow was seen for the Allplex assay; therefore, this assay was the preferred assay in our laboratory.

### Workflow optimization using Allplex SARS-CoV-2 assay and Hamilton STARlet

**Fig 3:**
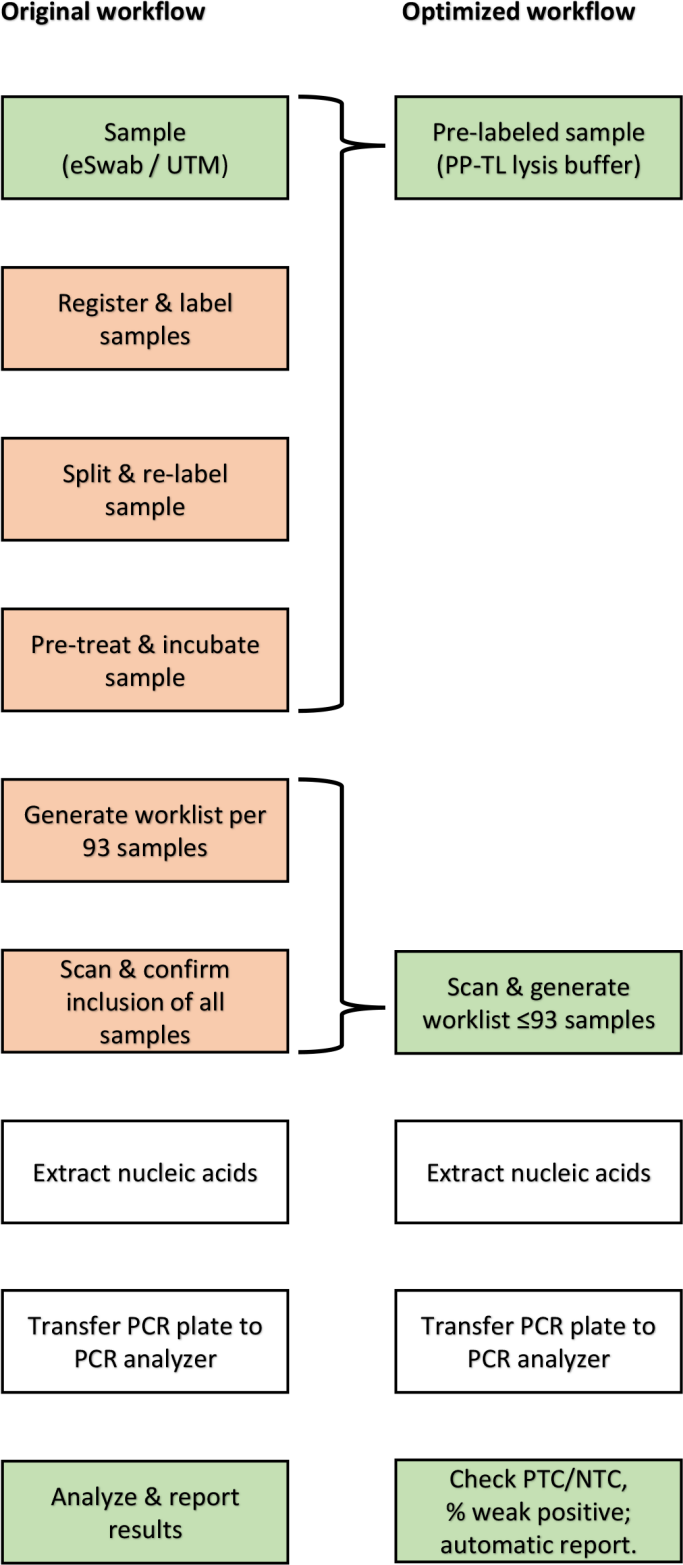
Overview of the initial workflow (left) and the desired workflow (right). Each step was analyzed with respect to what value each step of the diagnostic workflow added for the patient. Steps that could be optimized are colored orange. Process steps in green are considered optimized or optimal.

Initially, the Allplex SARS-CoV-2 assay was implemented in the laboratory similar to existing workflows: every sample is manually registered, checked, and pre-treated prior to testing. Each sample was prioritized based on patient characteristics (e.g., clinical case, health care worker, etc). However, this traditional approach can impact the productivity of the lab and cause prolonged turnaround times (15–17). To streamline the workflow, the workflow was visualized, and opportunities for improvement were identified (Fig. 3, orange squares). Options to optimize the workflow include the following:

1. The use of pre-labeled tubes with lysis buffer to preserve and pre-treat respiratory swabs, allowing for rapid registration and processing upon arrival at the laboratory.
2. Switching from priority-driven scheduling to first-come-first-served principle, allowing for a stringent, batch-wise processing schedule.
3. Replacing chemical pre-treatments with heat inactivation.
4. Replacing RT-PCR on purified nucleic acids with direct RT-PCR on clinical samples.
5. Simplifying the post-analysis process by focusing on selected quality controls rather than analyzing each RT-PCR.

Options (3) and (4) were not further explored by the laboratory in light of the significant reduction in sensitivity (20%–33%) especially in early cases with low loads of SARS-CoV-2, as previously reported (5, 7, 18–20).

### Validating pre-filled lysis tubes as a sampling container for SARS-CoV-2 testing

**Fig 4:**
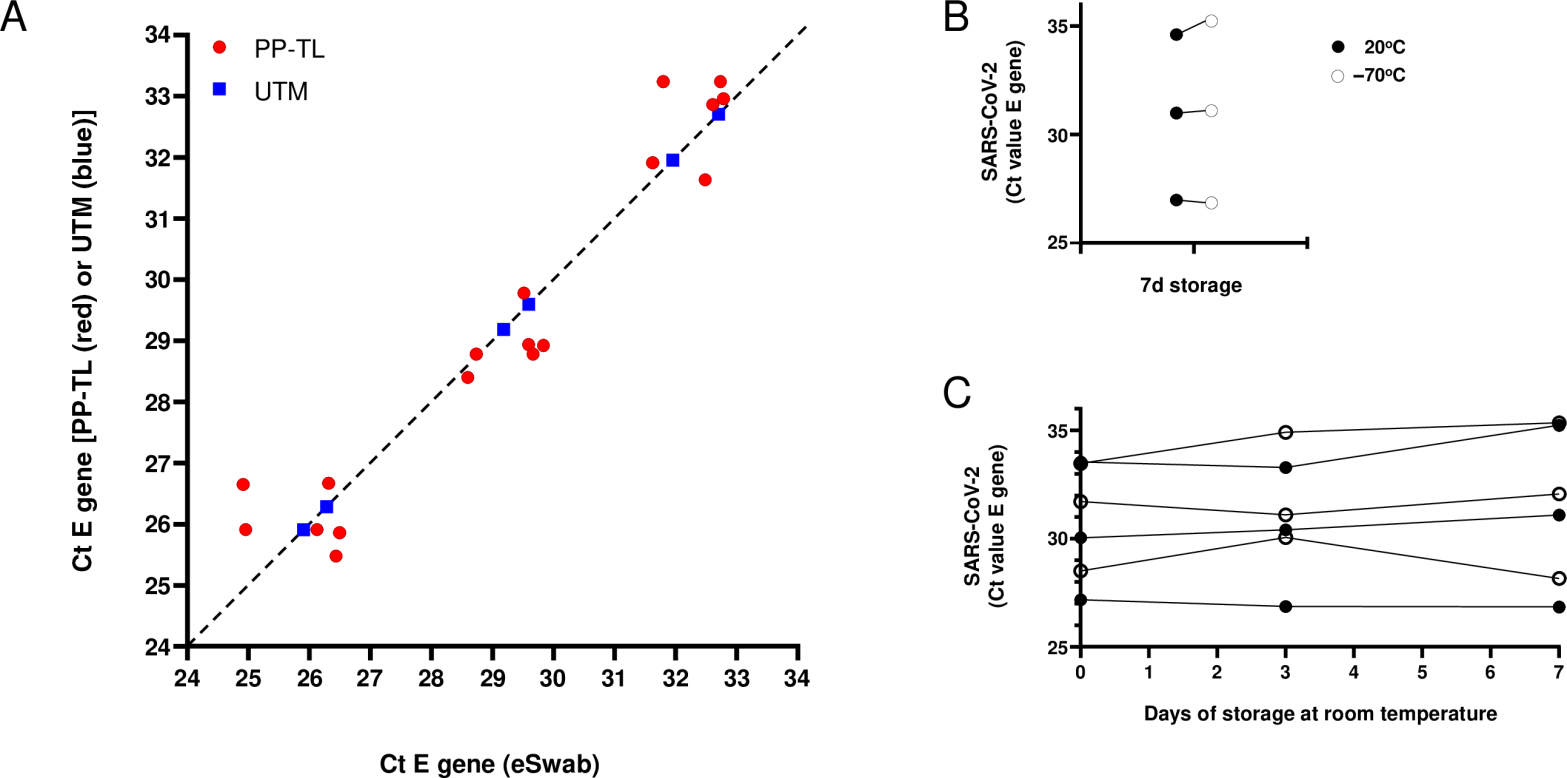
(A) Comparison of SARS-CoV-2 E gene Ct values obtained after spiking PP-TL+ (red) or UTM (blue) with SARS-CoV-2 relative to the corresponding Ct value seen in eSwab medium. The dotted line represents ideal comparison. *N* = 5 apparatuses. (**B**) Effect of temperature on SARS-CoV-2 Ct values in PP-TL+ after storage for 7 days at −70°C and at room temperature. (C) Effect of time on SARS-CoV-2 Ct values in PP-TL+ if samples are stored at room temperature and measured on days 0, 3, 7. *N* = 2 independent dilution series represented by either black or transparent dots.

First, to simplify the pre-analytical process, we verified the use of PP-TL+ as sampling method. PP-TL+ contains guanidine hydrochloride (15%–25%; i.e., 1.6–2.6 M), which is virucidal and inactivates nucleases (21). Using eSwab as the gold standard, we evaluated both PP-TL+ and UTM relative to eSwab. Spiking experiments did not show qualitative differences between the three sampling media (Fig. 4A). Importantly, the use of PP-TL+ enabled the storage of clinical materials up to 7 days at room temperature (Fig. 4B and C).

Strikingly, within the first week post-PP-TL+ implementation, samples in PP-TL+ were observed to have lower Ct values for the N gene compared with the Ct values for E and RdRp/S. This was not observed for samples collected in eSwab medium. Based on the work of Kim (22), we hypothesized that viral transcripts within scraped infected cells were conserved in PP-TL+, whereas these viral transcripts would be degraded by the cellular RNAses if collected in eSwab medium. Kim and colleagues found that the 3′ end of the SARS-CoV-2 genome (i.e., N gene) is much more abundantly transcribed than the upstream genes (e.g., ORF1ab, S, and E).

**Fig 5:**
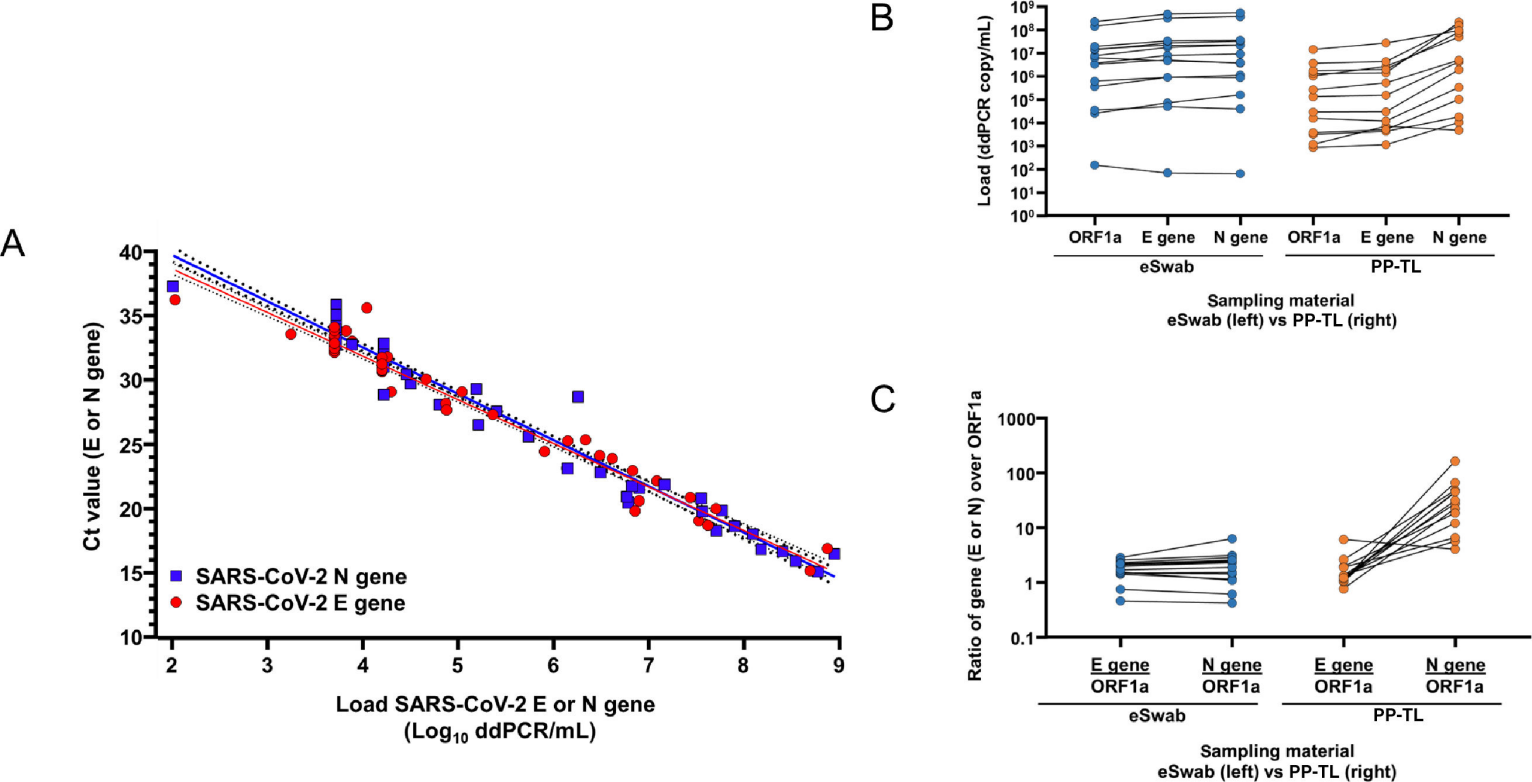
(A) Trendline and 95% CI of the N gene (blue) and E gene (red) concentration in the original material (ddPCR copies/mL); the equation for each is Log_10_ E gene load = 1/–3.396*(Ct E gene-45.45) and Log_10_ N gene = 1/–3.599*(Ct N gene-46,84). (B) Quantified numbers in clinical samples taken in eSwab (blue) or in PP-TL+ (orange). (C) Ratio of copy numbers for the E gene and N gene relative to the ORF1a.

We quantified the copy number of SARS-CoV-2 genes (ORF1ab, E, and N genes) by ddPCR (20), allowing us to (i) correlate the Ct values to absolute copy numbers in the original material (Fig. 5A) and (ii) compare copy numbers of ORF1a, E, and N in clinical samples collected in eSwabs and in PP-TL+ (Fig. 5B and C).

Quantification confirmed that clinical samples collected in PP-TL+ consistently contain more N gene transcripts than clinical samples collected in eSwab medium [mean ± SEM: N/ORF1a: 37.95 ± 11.63 (PP-TL+) *versus* 2.15 ± 0.39 (eSwab)]. The ratio of E/ORF1a was similar in both PP-TL+ and eSwab (1.77 ± 0.38 and 1.78 ± 0.18, respectively), indicating that the enhancing effect is N gene-specific.

### Validating the Allplex SARS-CoV-2 assay for deep respiratory samples

Second, we wished to use this workflow for deep respiratory materials (e.g., sputa and bronchoalveolar lavage). Deep respiratory materials initially were processed once a day using a labor-intensive, manual workflow. Automation was not possible due to the high viscosity of deep respiratory materials. Liquefaction of sputa by dithiothreitol (DTT) pre-treatment (23) visibly liquified the materials. while SARS-CoV-2 detection was not affected (data not shown). Moreover, DTT pre-treatment reduced the fraction of inhibited samples from 1.4% (6/427) to 0.5% (1/196).

Third, we compared the detection of SARS-CoV-2 in deep respiratory material from COVID-19 cases and compared these results with the respiratory swab taken on the same day (preferably) or with a ≤2-day difference (Table 4). This resulted in a concordance of 78% and Cohens kappa of 0.56 (moderate agreement), similar to previous results by Gao (24).

**TABLE 4:** Comparison of SARS-CoV-2 positivity in deep respiratory materials and nasopharyngeal/oropharyngeal swabs from known SARS-CoV-2-positive patients

		Deep material		
		Positive	Negative	
Respiratory swab	Positive	27	7	34
	Negative	8	26	34
		35	33	

Moreover, during the first year of working with the semi-automatic workflow, we regularly reevaluated our risk assessment. Based on our experiences, we gradually adapted the stringency of the checks: moving from visually checking each RT-PCR plot to an overall check of internal and external controls in combination with a thorough check of a random selection once per 2 weeks. This approach sufficed to observe the introduction of the RdRp/S-amplification variant in our region in November 2021 (25). Simplifying the analysis process of each RT-PCR run reduced the time needed and enabled the use of less-skilled technicians.

### Lean management from sample processing to result reporting: impact on turnaround times

**Fig 6:**
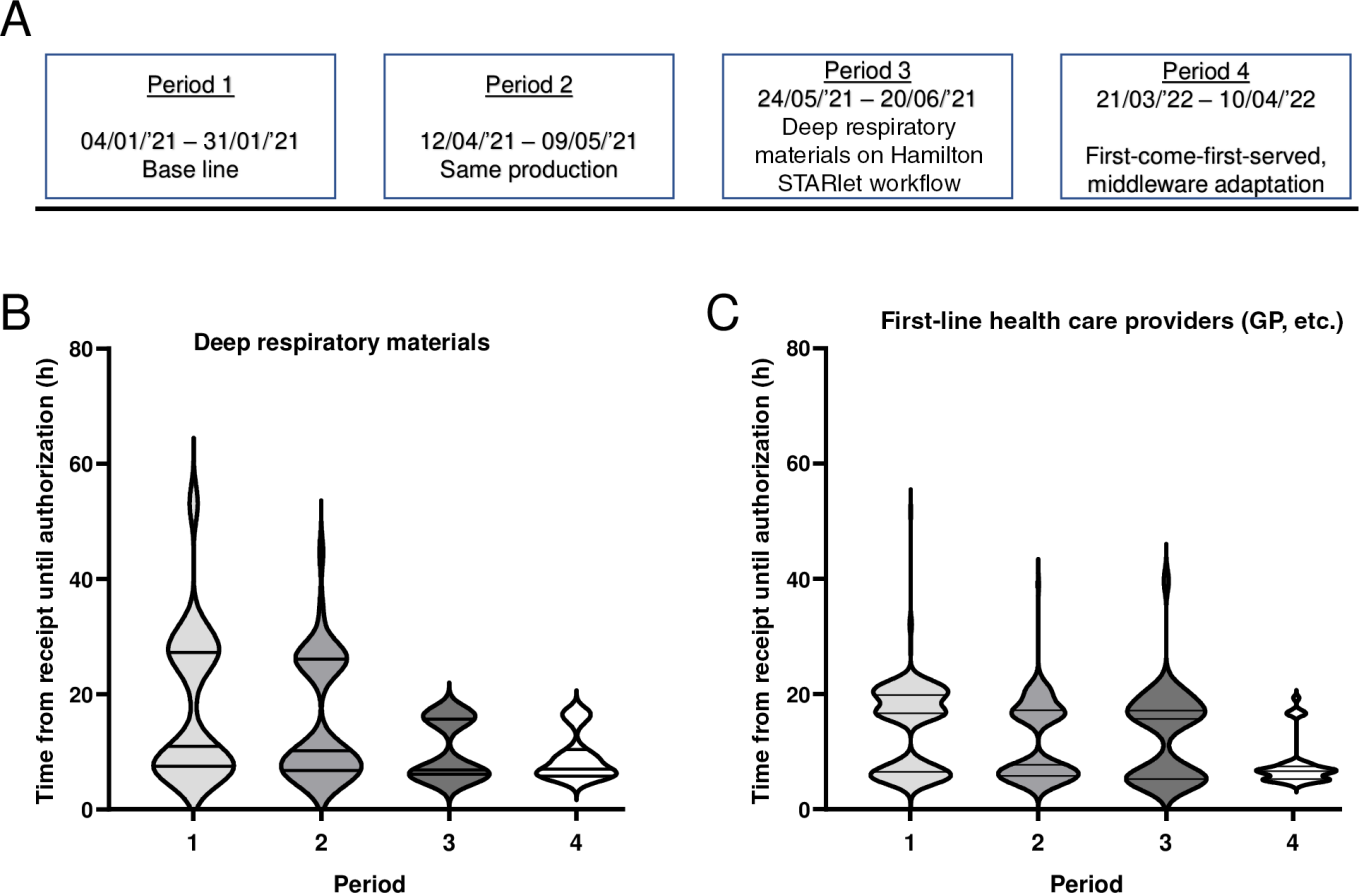
(A) Descriptive figure of the four selected periods wherein the turnaround time of the SARS-CoV-2 PCR was measured and the process optimizations that were implemented between the selected periods. (B and C) Violin plots of turnaround time for (**B**) deep respiratory materials (clinic’s) and (**C**) respiratory swabs (first-line health care providers; i.e., general practitioners and care homes). The violin plot shows the distribution of the turnaround times (length) combined with the frequency density for each possibility (width).

Finally, we adapted the laboratory middleware and removed the necessity to pre-book samples on specific working lists: the STARlet could scan and register the sample number, screen for SARS-CoV-2, and report the outcome of the RT-PCR to the laboratory information system, skipping the need for cross-checking samples with a predefined working list. Parallel to this, priority-driven testing was replaced with first-come-first-served, which removed the last potential bottlenecks in our workflow (Fig. 3 and 6A and B). We evaluated the effect of all optimizations by selecting four periods of 4 weeks each wherein productivity was similar (3,100/week, 2,750/week, 3,500/week, and 4,150/week, respectively; Fig. 6A). During this period, the laboratory was operational for 7 days per week.

For each period, the turnaround time (receiving samples until reporting) was retrieved from the laboratory information system (Fig. 6A and B). DTT pre-treatment of deep respiratory materials greatly reduced the turnaround time with 8.9 h {median [interquartile range (IQR)] period 1: 17.9 h (7.5–27.3 h) *versus* period 4: 9 h (5.8–10.4 h)} (Fig. 6A). Our first-line health care providers (general practitioners and nursing homes) benefitted from the introduction of the PP-TL+, which was loaded on the first available run without pre-treatment (median [IQR] period 1: 16.7 h [6.5–19.8 h] *versus* period 4: 6.6 h [5.2–7.4 h]) (Fig. 6B).

## DISCUSSION

We and others (26, 27) show that the Allplex SARS-CoV-2 assay is specific and does not cross-react with classic respiratory viruses. However, the sarbeco-broad Allplex E gene RT-PCR detects SARS-CoV-1. Though SARS-CoV-1 is currently not present in Europe, it should be considered in case of a solitary E gene with Ct <35. Importantly, our data show that medical laboratories should always verify or establish their own cutoff for the internal control (Supplemental data, Fig. S1A and B). Currently, the package insert applies Ct 42; however, we show that this pre-defined cutoff does not relate to the most reliable diagnostics as we showed that IC values above Ct 26 are related to poor diagnostic quality (Fig. S1A and B). We recommend laboratories to calculate their IC cutoff per lot number as the mean + 2 standard deviations of all IC values obtained in 10 runs.

The automated workflow using STARlet liquid handler in combination with either RAQ SARS-CoV-2 assay or Allplex SARS-CoV-2 assay is able to detect a SARS-CoV-2 load of 5,000 ddPCR copies/mL, matching previous reports (6, 27). Here, we observed that concentrations as low as 500 ddPCR copies/mL can be detected, albeit at lower probability of detection as signified by positivity in <3 targets (Table S1; Fig. 5A). Moreover, we show that the analytical sensitivity of SARS-CoV-2 assays can be increased by incorporating multiple targets (Tables 2 and 3; Table S3) as well as combining guanidine-containing sampling medium with specific N gene RT-PCRs (Fig. 5C). The use of multiple targets in an RT-PCR also prevents sudden loss-of-detection (25). Regarding the enhanced sensitivity of guanidine-containing buffers, we hypothesize that this phenomenon is caused by the inactivation of endogenous nucleases, resulting in preservation of the viral transcripts. Our results in clinical samples thus confirm the *in vitro* results of Kim (22) who found that, specifically, the N gene is abundantly transcribed within infected cells.

Enhanced sensitivity can be beneficial in the context of contact tracing. While not the focus of this study, one could argue that the ability to detect transcription may, in theory, differentiate those with an early, active infection (ratio N:ORF1a > 1) from those with post-COVID (ratio N:ORF1a ~ 1). On the other hand, enhanced sensitivity may also lead to clinically false-positive results (i.e., detecting SARS-CoV-2 in recent COVID-19 cases). To overcome this issue, laboratories will have to establish cutoffs based on, for example, single-target positives in a multiplex RT-PCR (28, 29) or the Ct value of the 90th percentile (14). Both conditions overlap at E gene ≥Ct 35 (i.e., <2,000 ddPCR copies/mL [Fig 5; Table S1]) and these samples are reported as weak positive for SARS-CoV-2. Carryover experiments show that cross-contamination is a possibility during processing of clinical samples and typically presents high Ct values. However, cases with early SARS-CoV-2 infections, or recently recovered from SARS-CoV-2, may also present with these Ct values, while the reproducibility at these Ct values can be poor. The requesting physician is advised to evaluate whether the patient may indeed have an early SARS-CoV-2 and thus pose a risk for other patients. To resolve this, physicians may request another SARS-CoV-2 test at 24–48 h after the previous sample.

We showed that minor modifications to the workflow enabled the analysis of deep respiratory materials on the STARlet workflow. However, the verification also showed that both materials are not to be considered the gold standard for exclusion of SARS-CoV-2. Deep respiratory materials had relatively low concordance with upper respiratory swab (our results [24]). Hence, screening of the upper respiratory tract may not suffice to exclude COVID-19 (30).

In summary, we described and validated several adaptations for semi-automatic SARS-CoV-2 screening resulting in significantly reduced turnaround times for deep respiratory materials (−9 h) and upper respiratory swabs (−10 h). Value stream mapping and risk assessment are ideal tools to identify opportunities for work standardization, thus reducing the overall analysis time as well as the dependency on highly skilled technicians.

## MATERIALS AND METHODS

### External quality assurance (EQA) samples

EQA samples were obtained from Quality Control for Molecular Diagnostics (QCMD, Edinburgh, UK) and the National Institute for Public Health and the Environment (Bilthoven, the Netherlands).

### Clinical samples

Clinical samples are collected from routine diagnostics conducted by our laboratory (Laboratory for Medical Microbiology and Immunology, Velp, the Netherlands). To test the clinical performance of the workflows and assays in this study, a random selection of nasopharyngeal/oropharyngeal swabs in 1 mL of UTM (Mediaproducts BV, Groningen, the Netherlands) or 1 mL of liquid Amies fluid (eSwab, Copan, Italy) or 2 mL of PP-TL+ (MolGen, Veenendaal, the Netherlands) was included. Similarly, remaining aliquots of deep respiratory materials (sputa, bronchial secretion, and bronchoalveolar lavage) were included.

### Sample preparation

Swabs in eSwab or UTM are pre-treated by mixing 400 µL of material with 400 µL of MP96 external lysis buffer (Roche) and left to incubate for at least 30 min prior to further processing. Swabs in PP-TL+ do not require pre-treatment.

Deep respiratory materials are liquified by mixing 400 µL of clinical material, 40 µL of 1M DTT (Merck, Darmstadt, Germany), and 360 µL of Tris-EDTA buffer (TE), pH 7.4, Bioultra (Merck, Darmstadt, Germany). Samples are shaken for 30 min at room temperature in a table shaker. Two aliquots are prepared from the liquified materials: one aliquot is processed as described above for eSwab/UTM (“pure”) and one part is one on four diluted with TE (80 and 320 µL, respectively) prior to further processing as described above for eSwab/UTM. In principle, the results obtained on the pure fraction are reported, unless inhibition is seen. In that case, the results of the diluted fraction are reported.

### Manual workflow

Nucleic acids were extracted on an easyMag (bioMérieux, France) using 200 µL of input and 100 µL of output. RT-PCR was carried out on an Applied Biosystems Fast 7500 (Thermo Fisher, Waltham, MA, USA) PCR machine in standard run mode using the SARS-CoV E Sarbeco assay (8) with TaqMan Fast Virus 1-Step Master Mix (Thermo Fisher, Waltham, MA, USA). Each RT-PCR reaction consisted of 10 µL of eluate and 20 µL of RT-PCR mix. Cycling conditions were 55°C for 10 min, followed by 94°C for 3 min then 45 cycles of 95°C for 15 s and 58°C for 30 s.

Controls consisted of SARS-CoV-2 as an external positive control, blanco extraction as an external negative control, and phocine herpes virus (European Virus Archive, Rotterdam, the Netherlands) as an internal extraction control. Samples that did not meet quality requirements were diluted 1:4 and processed as described above. If the sample failed twice to meet quality requirements, they are reported as “undetermined.”

### Allplex SARS-CoV-2 assay

Clinical materials are processed according to manufacturer’s instructions, using the STARMag Universal Extraction kit (Seegene, Seoul, South Korea) on the STARlet robotic platform (Hamilton, Reno, Nevada, USA). Each extraction required 200 µL of input for 100 µL of output. Each RT-PCR reaction consisted of 5 µL of eluate and 15 µL of RT-PCR mix. Cycling conditions were 50°C for 20 min, followed by 95°C for 15 min then 45 cycles of 95°C for 10 s, 60°C for 15 s, and 72°C for 10 s.

Controls included in the assay kit consisted of SARS-CoV-2 as an external positive control, Milli-Q water as an external negative control, and MS2 bacteriophage as an internal extraction control. Samples that did not meet quality requirements were retested once on the original tube. If the sample failed twice to meet quality requirements, they are reported as “undetermined.”

### RAQ SARS-CoV-2 Assay

Nucleic acids were extracted from clinical materials as described above using the Universal Extraction kit (Seegene). However, the eluates were used to set-up PCR reactions with the RAQ SARS-CoV-2 assay (PathoFinder, Maastricht, the Netherlands) using the open modus of the STARlet system. Each RT-PCR reaction consisted of 5 µL of eluate and 20 µL of RT-PCR mix. Cycling conditions were 50°C for 10 min, followed by 95°C for 1 min then 40 cycles of 95°C for 10 s and 60°C for 60 s.

Controls included in the assay kit consisted of SARS-CoV-2 as an external positive control, Milli-Q water as an external negative control, and MS2 bacteriophage as an internal extraction control.

### Middleware

Analysis and data processing of the manual and the STARlet Seegene workflow were performed using a middleware software referred to as FlowGO (LabHelp Labautomation, Bladel, the Netherlands). FlowGO arranges the communication between the Laboratory Information System and STARlet workflow and provided additional analysis of internal and external controls as well as result calling. It supports both the priority-driven workflow (original bi-directional) as well as the availability-driven (optimized, uni-directional) workflow.

### BioFire RP 2.1*plus*

Upper respiratory swabs in eSwab of UTM were processed using the BioFire RP2.1plus assay (bioMérieux, France) according to manufacturers’ instructions. Of the 24 targets, the laboratory validated the following pathogens: influenza virus A, influenza virus B, respiratory syncytial virus, human metapneumovirus, adenovirus, parainfluenzavirus (types 1–4), human endemic coronaviruses (OC43, NL63, 229E, and HKU-1), and rhinovirus/enterovirus (combined detection).

### GeneXpert rapid tests

Upper respiratory swabs in eSwab of UTM were processed using the GeneXpert (Cepheid, Sunnyvale, CA, USA) Xpert Xpress SARS-CoV-2, SARS-CoV-2 *plus*, SARS/Flu/RSV, and SARS/Flu/RSV *plus* assays as per manufacturer’s instructions. The initial tests detected only nucleocapsid (N2) and envelope (E) genes, whereas the *plus* versions also detected the RdRp gene.

### Droplet digital PCR

Droplet digital PCR was conducted on STARlet eluates (*N* = 29), as described before (20). Results were verified using WHO reference standard at 5,000 and 15,800 ddPCR copies/mL of the E gene (*N* = 24 each).

## Data Availability

The data sets used and/or analyzed during the current study are available from the corresponding author on reasonable request.

## SUPPLEMENTAL MATERIAL

Figure S1 spectrum.03296-23-s0001.pdf Establishment of an accurate cut-off for the internal control of the Allplex SARS-CoV-2 assay.

Supplemental tables spectrum.03296-23-s0002.docx Tables S1 to S5 and legend for Figure S1.

ASM does not own the copyrights to Supplemental Material that may be linked to, or accessed through, an article. The authors have granted ASM a non-exclusive, world-wide license to publish the Supplemental Material files. Please contact the corresponding author directly for reuse.
