# Supplementary figures and images for: Optimization of the STARlet workflow for semi-automatic SARS-CoV-2 screening of swabs and deep respiratory materials using the RealAccurate Quadruplex SARS-CoV-2 PCR kit and Allplex SARS-CoV-2 PCR kit
Source: Microbiol Spectr. 2024 Jan 9;12(2):e03296-23. doi: 10.1128/spectrum.03296-23 (PMC10846099; doi:10.1128/spectrum.03296-23)

A

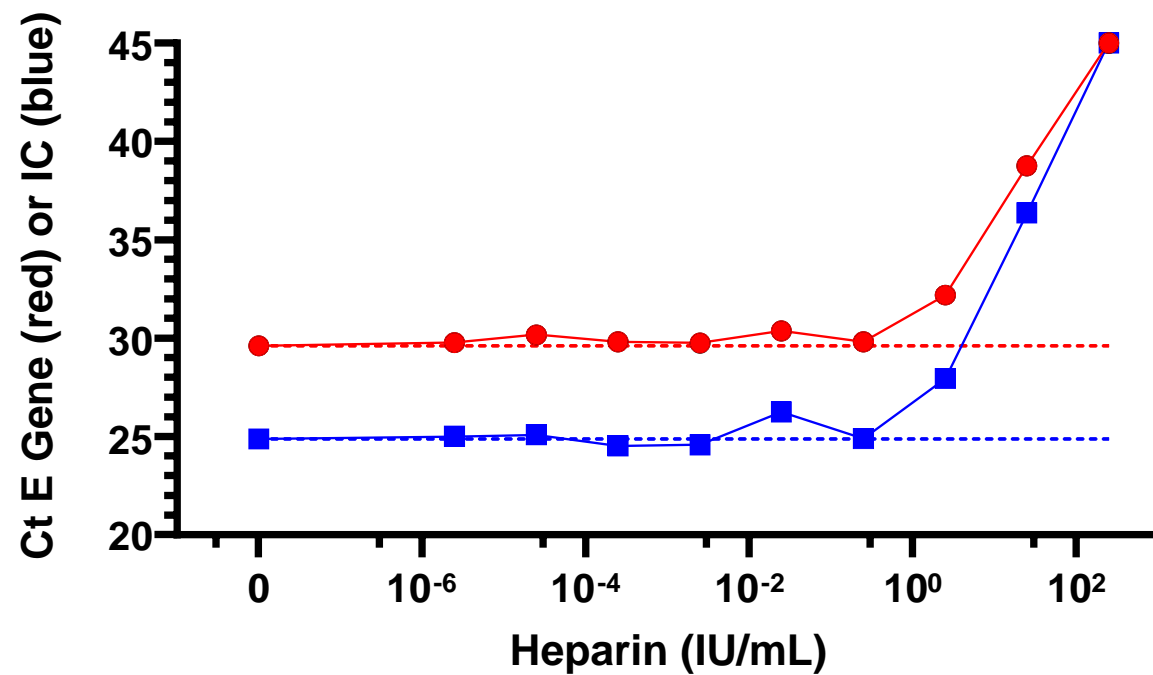

B

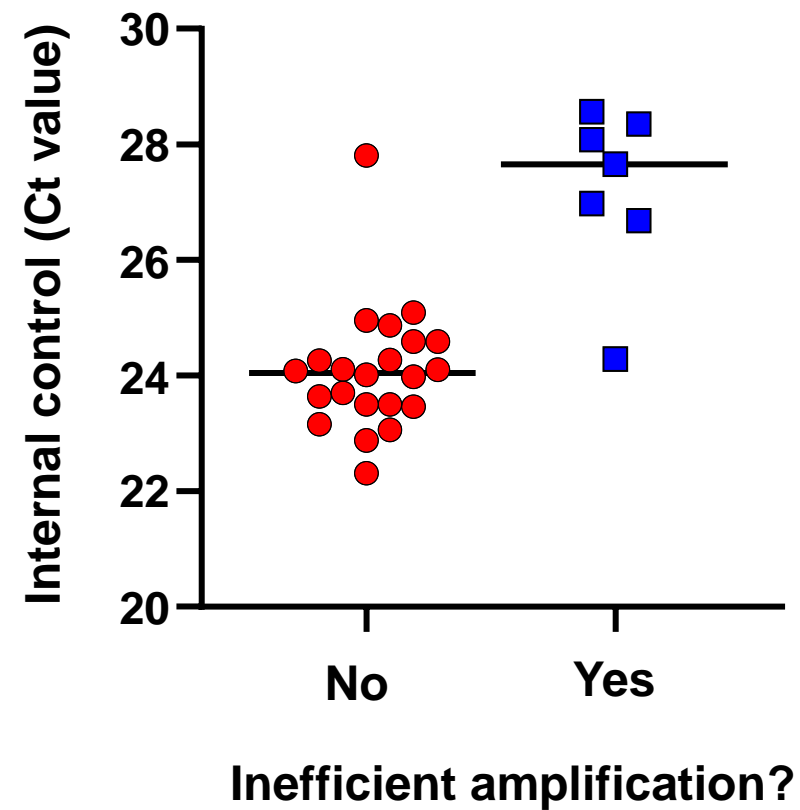

Supplement: Figure S1 — Establishment of an accurate cut-off for the internal control of the Allplex SARS-CoV-2 assay. [file spectrum.03296-23-s0001.pdf]
