# Supplementary material for: Optimization of the STARlet workflow for semi-automatic SARS-CoV-2 screening of swabs and deep respiratory materials using the RealAccurate Quadruplex SARS-CoV-2 PCR kit and Allplex SARS-CoV-2 PCR kit
Source: Microbiol Spectr. 2024 Jan 9;12(2):e03296-23. doi: 10.1128/spectrum.03296-23 (PMC10846099; doi:10.1128/spectrum.03296-23)
Supplement: Supplemental tables — Tables S1 to S5 and legend for Figure S1. [file spectrum.03296-23-s0002.docx]

Supplemental information to:

Optimization of the STARlet workflow for semi-automatic SARS-CoV-2 screenings on swabs and deep respiratory materials using the RealAccurate® Quadruplex SARS-CoV-2 PCR kit and Allplex SARS-CoV-2 PCR kit

Jacky Flipse^1^, Angelino T. Tromp^1,2^, Danique Thijssen^1^, Nicole van Xanten-Jans-Beken^1^, Roy Pauwelsen^1^, Harmen J. van der Veer^3,4^, Juliëtte M. Schlaghecke^5^, Caroline M.A. Swanink^1^

1. Laboratory for Medical Microbiology and Immunology, Rijnstate Hospital, Velp, the Netherlands
2. present address: Medical Laboratory Services, Willemstad, Curaçao
3. Laboratory of Chemical Biology, Dept. Of Biomedical Engineering, Eindhoven University of Technology, Eindhoven, the Netherlands
4. Institute for Complex Molecular Systems, Eindhoven University of Technology, Eindhoven, the Netherlands
5. Research Group Applied Natural Sciences, Fontys University of Applied Sciences, Eindhoven, the Netherlands

Table S1: Performance of the Allplex SARS-CoV-2 assay per viral load

|  |  | External Quality Assurance (SARS-CoV2-) † | | |
| --- | --- | --- | --- | --- |
|  |  | >1·10^4^ ddPCR/mL  (n=36) | 5·10^2^ – 1·10^4^ ddPCR/mL (n= 17) | <5·10^2^ ddPCR/mL  (n=10) |
| Allplex SARS-CoV-2 | All 3 targets positive | 36 | 12 | 4 |
|  | <3 targets positive |  | 5 | 2 |
|  | Negative |  |  | 4 |

The Allplex SARS-CoV-2 assay is a multiplex RT-PCR detecting four SARS-CoV-2 genes with three dyes: E, N, RdRp and S, with the latter two combined in one dye.

† EQA samples are derived from QCMD and the National institute for Public Health and the Environment (Bilthoven, the Netherlands). Samples include the following SARS-CoV-2 types; B.1, B.1.1.7; B.1.117; B.1.351; B.1.525; B.1.526; B.1.529 (BA.1; BA.2); B.1.617.2; P.1.

Table S2: The Allplex SARS-Cov-2 assay does not cross-react with other typical respiratory pathogens

|  |  | Second pathogen | | | | | | | | | |
| --- | --- | --- | --- | --- | --- | --- | --- | --- | --- | --- | --- |
|  |  | AdV | hCoV | RhV/EnV | Bparap | InflA | PIF3 | PIF4 | RSV | hMPV | SARS-CoV-2 |
| First pathogen | AdV | 1 | 1 | 2 | 1* | 1* |  |  | 1* |  |  |
|  | hCoV | 1 | 3 | 1† |  |  |  |  | 1† |  |  |
|  | RhV/EnV | 2 | 1† | 16 |  | 1 |  |  | 3 | 1 |  |
|  | Bparap | 1* |  |  |  |  |  |  |  |  |  |
|  | InflA | 1* |  | 1 |  | 4 |  |  |  |  |  |
|  | PIF3 |  |  |  |  |  | 2 |  |  |  |  |
|  | PIF4 |  |  |  |  |  |  | 2 |  |  |  |
|  | RSV | 1* | 1† | 3 |  |  |  |  |  |  |  |
|  | hMPV |  |  | 1 |  |  |  |  |  | 2 |  |
|  | SARS-CoV-2 |  |  |  |  |  |  |  |  |  |  |
|  | Total | 5 | 5 | 24 | 1 | 6 | 2 | 2 | 5 | 3 | 0 |

This table represents the clinical materials tested with the Allplex SARS-CoV-2 assay; most clinical materials test positive for one or two pathogens. The number of materials with multiple pathogens can be deduced from this table. Only two materials contained more than pathogens. These are denoted with * or †. AdV, Adenovirus; hCoV, endemic human coronaviruses (229E, OC43, HKU-1, NL63); RhV/EnV, Rhinovirus/Enterovirus; Bparap, Bordetella parapertussis; InflA, Influenza virus type A; PIF3, parainfluenzavirus type 3; PIF4, parainfluenzavirus type 4; RSV, respiratory syncytial virus; hMPV, human Metapneumovirus.

Table S3: Performance of the GeneXpert and Allplex SARS-CoV-2 assays

|  |  | GeneXpert  SARS-CoV-2 assay | | | GeneXpert  SARS-CoV-2 assay *plus* | | | GeneXpert  SARS/Flu/RSV | | GeneXpert  SARS/Flu/RSV *plus* | |
| --- | --- | --- | --- | --- | --- | --- | --- | --- | --- | --- | --- |
|  |  | E+ | E-, Gx+ | Gx- | E+ | E-, Gx+ | Gx- | + | - | + | - |
| Allplex SARS-CoV-2 | E+ | 91 | 3 | 3 | 52 | 1 | 1 | 54 | 1 | 50 | 1 |
|  | E-, SARS+ | 2 | 6 | 3 | 2 | 0 | 0 | 0 | 0 | 3 | 0 |
|  | Negative | 9 | 5 | 3* | 7 | 0 | 0 | 7 | 0 | 5 | 4* |

*Gx+: The GeneXpert assay tested positive for genes other than the E gene (i.e. N2 and/or RdRp). * Samples concern samples with known SARS-Cov-2 viral load <320 E gene ddPCR copies/mL.*

Table S4: retrospective verification of Real Accurate Quadruplex (RAQ) SARS-CoV-2 assay RdRp gene relative to Allplex SARS-CoV-2 assay E gene

|  |  | Allplex SARS-CoV-2 (E gene) | | |
| --- | --- | --- | --- | --- |
|  |  | E Ct <35,0 | E Ct ≥35,0 | Negative |
| RAQ SARS-CoV-2  (RdRp) | Positive | 112 | 0 | 0 |
|  | Negative | 5 | 4 | 513 |

*None of the samples were invalid due to the presence of inhibitory factors*

Table S5: reproducibility data based on the N gene Ct values in Seegene SARS-CoV-2 assay and Real Accurate Quadruplex (RAQ) SARS-CoV-2 assay

|  |  | Allplex SARS-CoV-2 | | RAQ SARS-CoV-2 | |
| --- | --- | --- | --- | --- | --- |
| SARS-CoV-2 concentration (copies/mL) | | 5000 | 15.823 | 5000 | 15.823 |
| Probability of Detection | | 100% (24/24) | 100% (24/24) | 100% (24/24) | 100% (24/24) |
| Ct values (mean ± standard deviation) | |  |  |  |  |
| N gene | | 33.86±0.88 | 31.80±0.48 | 36.74±1.42 | 34.33±0.94 |
| RdRp/S (Allplex) / RdRp (RAQ) | | 33,93±0.70 | 32,11±0.37 | 35,96±1,14 | 33,48±0.80 |
| E gene (Allplex) | | 32,99±0.51 | 31,10±0.28 | N/A | N/A |

*None of the samples were invalid due to the presence of inhibitory factors. Reproducibility was determined by testing 6 replicates of the same virus stock per day over 4 days (N=24 total). Targets included in the assays are: the Allplex assay includes E gene, N gene, and one probe dye for both RdRp and S genes. The Real Accurate Quadruplex (RAQ) assay includes RdRp gene and N gene.*

Figure S1: Establishment of an accurate cut-off for the Internal control of the Allplex SARS-CoV-2 assay

Figure S1A: The concentration-dependent effect of heparin on the Ct value of the SARS-CoV-2 E gene (red circles) and the internal control (MS2 bacteriophage; blue squares). The dashed line shows the base line Ct value in absence of any inhibitors.

Figures S1B: Ct values of the Allplex SARS-CoV-2 internal control (bacteriophage MS2) in pure samples containing inhibitory factors (blue squares) and in those without (red circles); if diluting the sample resulted in decreased Ct values, this was considered indicative of inefficient extraction and/or amplification due to inhibitory factors in the sample.

Materials and methods for Figure S1:

Figure S1A: Heparin was used as a PCR-inhibitory compound to define the cut-off of the IC that separates reliable PCR results from potentially non-reliable PCR reactions. A fixed amount of SARS-CoV-2 was added to eSwab medium and aliquoted over ten samples, each containing the same amount of SARS-CoV-2, corresponding with E gene Ct 30. This is defined as the baseline (red dashed line in Figure S1A). Subsequently, a dilution series of heparin (Greiner Bio One, Frickenhausen, Germany, ref: 484523) was made in eSwab medium. A small amount of this heparin solution was added to the tubes resulting in a final concentration of 252 IU/mL – 2.5·10^-6^ IU/mL (Figure S1A). Inhibition was defined as a Ct of 31.02 or above (i.e. Ct 30 + twice the standard deviation of 0.51Ct). This threshold was crossed at heparin concentrations of ≥2,5IU/mL. Those conditions were defined as inhibited PCR reactions. In non-inhibited PCR reactions, the highest Ct value of the IC was 26.3. In case of inhibited PCR reactions, the lowest Ct value of the IC was Ct 28.

Figure S1B: Deep respiratory materials (e.g. sputa, bronchial secretion, bronchoalveolar lavage) are samples known to contain inhibitory factors. Hence, these are pre-treated with DTT, as described in the main text, and subsequently either processed directly or processed after diluting the material 1 on 4 (i.e. 80μL pre-treated material on 320μL TE buffer). This allows for retrospective analysis of paired samples (pure and diluted) with at least one SARS-CoV-2 positive sample. Here, inhibition of the SARS-CoV-2 PCR is defined as less than 1.4Ct difference in SARS-CoV-2 Ct values of the paired samples. The cut-off of $\leq$1.4Ct is based on +2.3Ct (5-fold dilution) minus twice the standard deviation (0.53Ct). Samples with inefficient amplification of SARS-CoV-2 were considered inhibited and are plotted in Figure S1B (Blue squares). Samples without indication for inhibition are plotted as red circles (Figure S1B), showing that inhibited samples generally had IC values >Ct 26, in line with lithium heparin-induced inhibition (Figure S1A).
